# Numerical Study on the Acoustic Transmission Performance of New Hierarchical Honeycomb Sandwich Panel

**DOI:** 10.3390/ma19153222

**Published:** 2026-07-28

**Authors:** Boyan Zhou, Qiang He

**Affiliations:** School of Mechanical Engineering, Jiangsu University of Science and Technology, Zhenjiang 212000, China; boyan_zhou2026@163.com

**Keywords:** sound insulation, vibration, sandwich panels, honeycomb, hierarchical structure

## Abstract

A novel hierarchical honeycomb structure is proposed as the core layer of sandwich panels, replacing the hexagonal vertices with other shapes. Its vibration and sound insulation performance are further discussed. Structural acoustic finite element analysis methods were used to simulate the natural frequency, sound transmission loss (STL), and sound pressure distribution within the acoustic domain of the sandwich panels. Within the given simulation parameter range, the sound insulation efficiency of the new hierarchical honeycomb sandwich panel was significantly improved, and the triangular vertex configuration exhibited the best noise reduction ability. By varying the vertex size and the dimensions of the units, the sandwich panels’ vibration reduction and noise insulation capabilities can be further optimized. The average sound transmission loss (STLo) for hierarchical parameter (the ratio of the vertex edge length to the wall length) λ = 0.4 increases by 17.2% compared to λ = 0.2, greatly improving sound reduction efficiency. When the size of the honeycomb unit is small, the sandwich panel exhibits enhanced acoustic transmission loss within the resonance frequency range. The hierarchical honeycombs after vertex triangle rotation show an STLo level of around 43.08–44.24 dB. The influence of vertex triangle rotation on the STLo of hierarchical honeycomb is closely related to the hierarchical parameters, with STLo increasing by 14.8% for λ = 0.2 and 4% for λ = 0.3, while the opposite phenomenon occurs when λ is 0.4. The research results provide valuable insights into improving the vibration reduction and sound insulation performance of honeycomb sandwich panels within the target frequency range by introducing hierarchical features. However, related engineering applications need to rely on subsequent experimental verification.

## 1. Introduction

The honeycomb sandwich structure is composed of thin panels and lightweight porous core layers, which can achieve excellent bending stiffness, yield resistance, and energy absorption characteristics under low-surface-density conditions. It has been widely used in fields such as aerospace, rail transportation, shipbuilding, automotive transportation, and civil protection. At present, research on honeycomb sandwich structures both domestically and internationally is relatively systematic, mainly focusing on lightweight load-bearing designs [1], multifunctional integration [2], and dynamic impact protection [3,4]. Experimental and theoretical research on key scientific issues such as dynamic crushing behavior [5], static stiffness and strength characteristics [6], explosive load responses [7], local impact damage mechanisms [8], and load-bearing bending mechanics [9] is still being continuously advanced.

With the continuous improvement of technical requirements for lightweight equipment in terms of ride comfort, stealth performance, and service reliability, the research focus on honeycomb sandwich structures has gradually extended from single mechanical load-bearing performance to functional fields, such as structural vibration suppression, sound insulation, and noise reduction. Ruzzene et al. [10] conducted research on the vibration characteristics and acoustic radiation laws of honeycomb truss core beams and confirmed that the topology configuration of the core can significantly change the natural modes and acoustic radiation efficiency of the structure. Griese et al. [11] further elucidated the regulatory mechanisms of honeycomb core geometric parameters on sound insulation transmission performance. Arunkumar et al. [12] and Meng et al. [13] explored optimization and improvement strategies for low-frequency vibrations and acoustic performance from multiple dimensions.

However, the traditional conventional honeycomb structure has a relatively single and fixed-cell configuration, which presents a significant trade-off between low-frequency sound insulation, broadband noise reduction, and lightweight load-bearing requirements, making it difficult to achieve comprehensive performance synergy improvement. To overcome this performance bottleneck, researchers have introduced unconventional cell configurations, negative Poisson’s ratio superstructures, acoustic metamaterials, and topology optimization techniques into the innovative design of sandwich core layers. Existing studies on perforated lattice truss cores [14], differentiated Poisson’s ratio cellular core layers [15], gradient negative Poisson’s ratio annular structures [16], and star-shaped negative Poisson’s ratio cells [17] have shown that new cell configurations can effectively improve the sound insulation and noise reduction performance of sandwich structures in specific frequency bands by regulating the structural resonance characteristics, elastic wave propagation paths, and interface impedance mismatch mechanisms. Luo et al. [18], Denli et al. [19], and Oliazadeh et al. [20] conducted structural acoustic collaborative optimization research on periodic sandwich structures and honeycomb sandwich panels, further improving the acoustic performance optimization design system. At the same time, studies confirmed that artificial microstructures can optimize the acoustic response characteristics of structures through equivalent medium-parameter designs and precise control of the wavefield [21,22,23]. Ciaburro et al. [24] conducted experiments integrating perforated panels with honeycomb structures, revealing notable improvements in sound absorption at specific frequencies. The above research provides a new technical approach for the vibration reduction and sound insulation design of honeycomb sandwich structures.

In order to further improve the mechanical performance of regular honeycomb designs, many researchers sought inspiration from nature and proposed hierarchical honeycomb structures. Ajdari et al. [25], Haghpanah et al. [26], and Oftadeh et al. [27] pioneered fundamental research on hierarchical honeycombs. It has been clarified that hierarchical parameters can effectively regulate the equivalent stiffness, yield mode, and deformation mechanism of structures. Relevant studies further verify that hierarchical honeycombs possess outstanding application potential in Poisson’s ratio regulation, thermal resistance improvement, and collaborative optimization of mechanical properties [28,29,30]. In the field of impact protection and energy absorption, Sun et al. [31] and He et al. [32] analyzed the explosion response and dynamic crushing law of hierarchical honeycomb sandwich panels and self-similar hierarchical honeycombs. Subsequent researchers extended the hierarchical design to a variety of novel porous configurations, such as re-entrant honeycombs [33], embedded reinforced honeycombs [34], Kresling origami honeycombs [35,36], hierarchical square honeycombs [37], bidirectional hierarchical honeycombs [38], and serial honeycombs [39], and systematically investigated their mechanical and energy-absorbing properties.

Existing studies on hierarchical honeycombs mainly focus on their quasi-static bearing capacity, dynamic impact response, and compressive performance improvement. Numerous studies have verified that the introduction of hierarchical features can significantly enhance the mechanical properties of honeycomb structures, which provides theoretical references for the application of novel honeycomb structures in vibration reduction and noise insulation. Nevertheless, studies concerning the acoustic functional design of hierarchical honeycombs remain relatively scarce at present. Zhou et al. [40] combined hierarchical honeycomb cores with micro-perforated sandwich panels and investigated the broadband sound absorption performance under high sound pressure levels. Wang et al. [41] proposed an acoustic metamaterial with hierarchical honeycomb cores and verified that coupled hierarchical architecture can effectively strengthen broadband sound absorption capacity. Miao et al. [42] established an equivalent single-layer model for sandwich panels with hierarchical diamond honeycomb cores, which offers an efficient modeling approach and parametric analysis strategy for complex hierarchical core structures. To expand the bandwidth, researchers have attempted to combine micro-perforated plates with periodic lattice structures, such as honeycombs [43,44,45]. Lu et al. [46] conducted research on the optimization of honeycomb-like acoustic metamaterials for railway noise control based on Kolmogorov–Arnold networks.

Replacing the vertices of the regular honeycomb with smaller hexagons, rectangles, or circles is one of the typical methods for introducing hierarchical characteristics. The mechanism of the influence of different vertex geometries (hexagonal, circular, or triangular) on sound insulation performance is still unclear. There is a lack of systematic parameterization research on how hierarchical parameters affect sound transmission loss (STL) and sound pressure distribution. In this paper, the acoustic transmission performance of hierarchical honeycombs is investigated.

The organization of the following sections in this paper is as follows: Section 2 provides a comprehensive overview of the novel hierarchical structure proposed in this study. In Section 3, we establish a structural acoustic finite element model and conduct validation of the model. Section 4 provides an in-depth analysis of the physical mechanism of sound insulation and compares hierarchical honeycomb units with different vertex geometries. Furthermore, the influence of vertex size, cell size, and triangle vertex angle on the vibration and sound insulation performance of hierarchical honeycomb is further studied in order to clarify the regulatory mechanisms of local hierarchical geometry on sound transmission behavior.

## 2. Modeling

**Figure 1:**
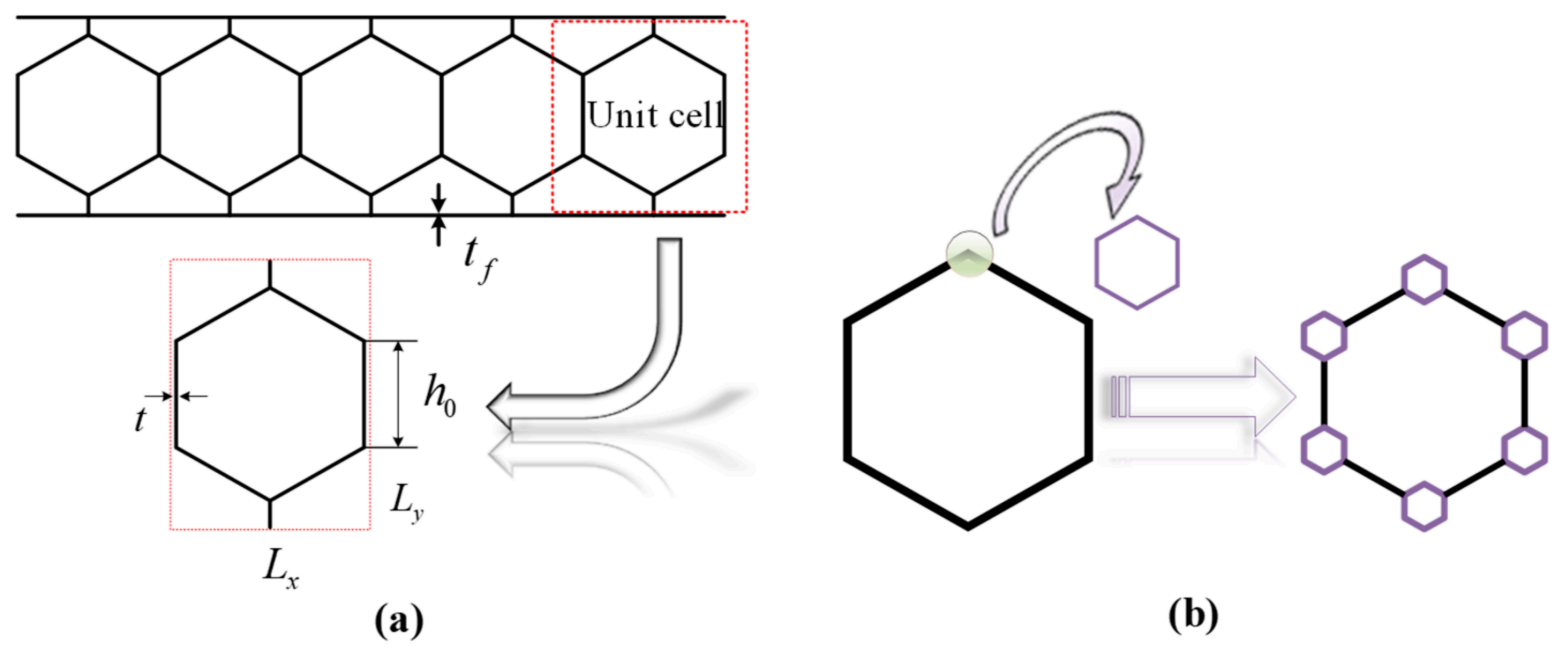
(**a**) Initial configuration of the sandwich panel and unit cell; (**b**) the vertices of the regular hexagonal honeycomb are replaced with hexagons.

As shown in Figure 1a, the sandwich panel consists of three layers, namely two panels and one honeycomb core. As a new type of honeycomb design, its feature is that the vertices of the regular hexagonal honeycomb structure are replaced with various shapes, resulting in a higher-order hierarchical honeycomb structure. The length of the panels is 2000 mm, with the core layer comprising 1 × 40 honeycomb units. Figure 1b depicts a representative unit where the vertices are replaced by hexagons. Figure 1a illustrates the geometric configuration of the honeycomb units, including the horizontal length $L_{x}$, the vertical dimension of the core layer $L_{y}$, the hexagon wall length $h_{0}$, the wall thickness t, and the panel thickness $t_{f}$. The following equations provide the relationships between the geometric parameters and unit dimensions.

(1) $$L_{x}=2h_{0}cos\theta$$

(2) $$L_{y}=2h_{0}(1+sin\theta )$$

The reference dimensions are determined based on the standard hexagon; thus, the angle θ is set at 30 degrees. Given that $L_{x}$ is specified as 50 mm, the corresponding values of $h_{0}$ and $L_{y}$ are calculated to be 28.87 mm and 86.6 mm, respectively. Consequently, the overall length of the sandwich panel aligns with the aforementioned measurement of 2000 mm.

**Figure 2:**
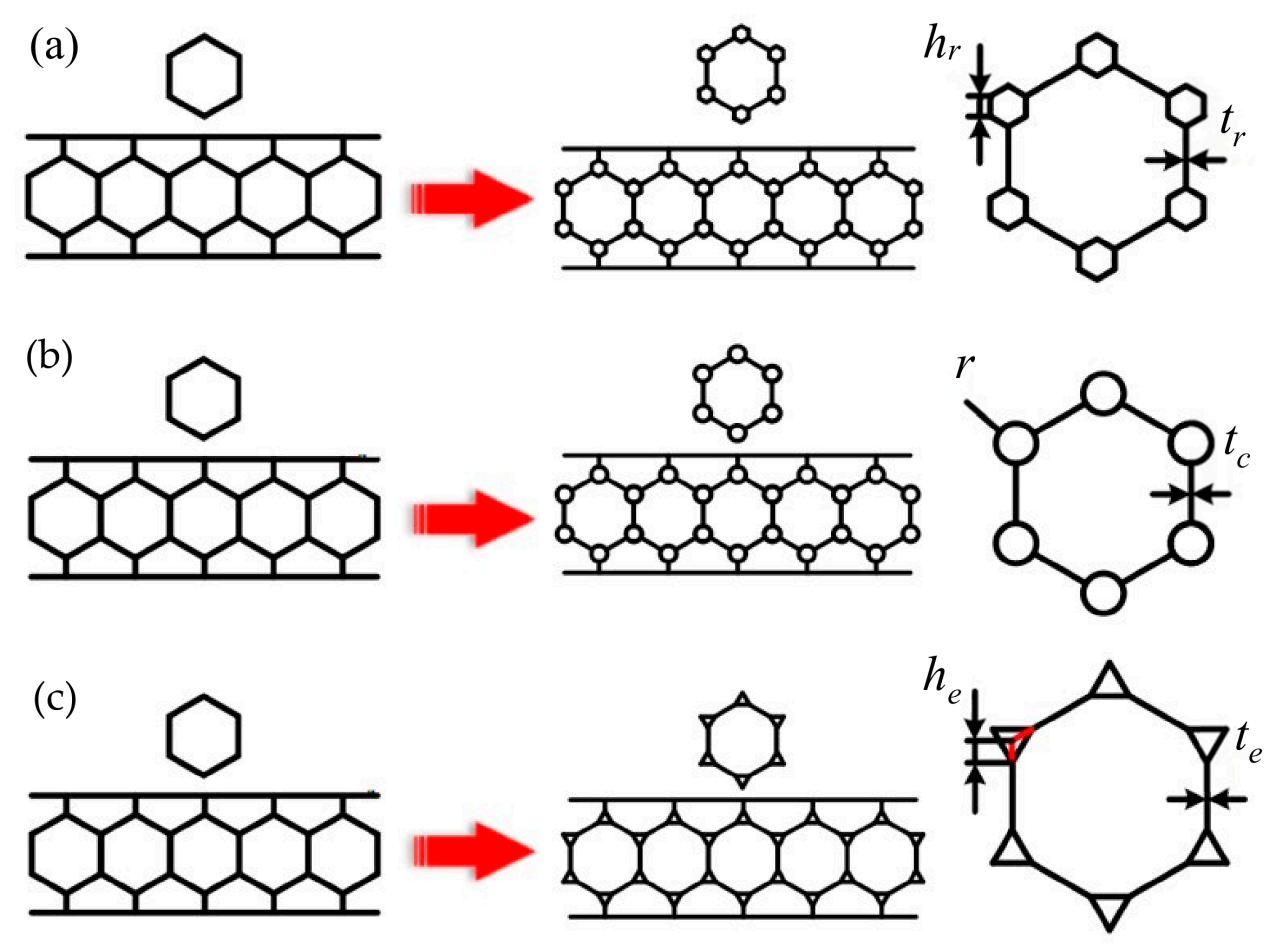
Schematic diagram of hierarchical honeycomb sandwich panels: (**a**) vertices as regular hexagons; (**b**) vertices as circles; (**c**) vertices as equilateral triangles.

As depicted in Figure 2, the vertices of the regular hexagonal honeycomb structure are replaced with hexagons, circles, and equilateral triangles, thus yielding three distinct hierarchical configurations. The edges of the hexagon, circle, and equilateral triangle are designated as $h_{r}$, r and${\;h}_{e}$, respectively, while the hierarchical parameter λ is explained as the ratio of the vertex edge length to $h_{0}$. In order to prevent overlap of the vertex units, the condition 0≤λ≤0.5 must be satisfied. As for the manufacturing of honeycomb structures, conventional production technology only produces some regular honeycomb structures. With the development of 3D printing, or additive manufacturing, the manufacturing of the aluminum samples with the novel cell configurations can be realized. The apparent density serves as a crucial parameter of the structure, which is calculated using the area proportion approach [47]. Through this approach, the mass located in the unit area is equivalent to that of the matrix material, allowing the apparent mass density of the regular honeycomb to be expressed as:

(3) $$\rho_{r}=\frac{2}{\sqrt{3}}\frac{t}{h_{0}}\rho_{s}$$

where $\rho_{s}$ refers to the density of the matrix material, and the relative density $\rho_{rel-r}$ of the regular honeycomb is expressed as:

(4) $$\rho_{rel-r}=\rho_{r}/\rho_{s}=\frac{2}{\sqrt{3}}\frac{t}{h_{0}}$$

The apparent density $\rho_{f}$ and the relative density $\rho_{rel-h}$ of the hexagonal hierarchical structure are outlined below:

(5) $$\rho_{f}=\frac{2(1+2\lambda )}{\sqrt{3}}\frac{t_{r}}{h_{0}}\rho_{s}$$

(6) $$\rho_{rel-h}=\rho_{f}/\rho_{s}=\frac{2(1+2\lambda )}{\sqrt{3}}\frac{t_{r}}{h_{0}}$$

Therefore, while maintaining the same relative density, it can be simplified to:

(7) $$t_{r}=\frac{t}{1+2\lambda }$$

Similarly, the formulas for calculating the wall thickness of the circular and triangular configurations are obtained as follows:

(8) $$t_{c}=\frac{t}{1-2\left(1-\frac{2}{3}\pi \right)\lambda }$$

(9) $$t_{e}=\frac{t}{1+2(\sqrt{3}-1)\lambda }$$

The baseline model M1 uses *t* = 2.5 mm consistently across all simulations. Consequently, for hierarchical structures with vertices shaped as regular hexagons, circles, and equilateral triangles, and with a hierarchical parameter set at 0.2, the wall thickness of the honeycomb units is determined to be 1.78 mm, 1.74 mm, and 2.09 mm, respectively. Detailed parameters of the honeycomb units are comprehensively presented in Table 1. Changing the size of hierarchical cells will alter the overall thickness of the cells. However, for these four structures, $t_{f}$ is 2.5 mm, $L_{x}$ is 50 mm, and the corresponding values of $h_{0}$ and $L_{y}$ are calculated to be 28.87 mm and 86.6 mm, respectively. It can be confirmed clearly that all compared configurations have an equivalent mass, relative density, and overall dimensions.

## 3. Numerical Simulation

### 3.1. Finite Element Model

**Figure 3:**
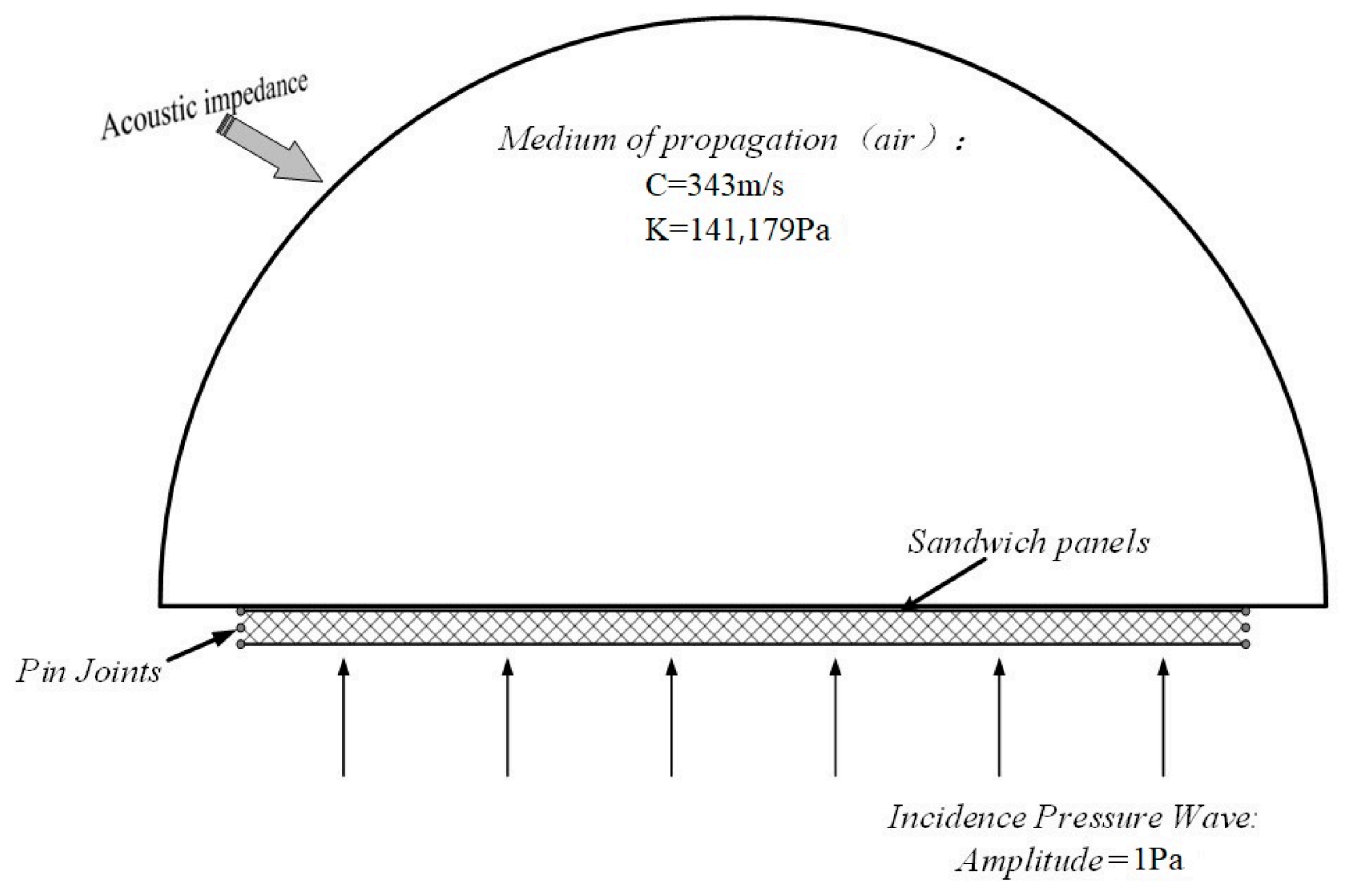
Acoustic FEM (finite element method) model for the sandwich panel structure.

The vibration and acoustic responses of different structural layouts of hierarchical honeycomb sandwich panels were numerically studied using the commercial finite element application ABAQUS 2022. As illustrated in Figure 3, an acoustic structural model was established to explore the vibrational sound characteristics of the sandwich panel, including stiffness control regions and resonance areas.

A two-dimensional quadratic beam element (B22) was used to model the sandwich panel. The air domain was represented by a semicircular area and was meshed using AC2D3. By using a mesh bias from the center of the semicircular domain to the edge, the element size gradually increased at the circular edge of the air domain. The finite element model comprises, in total, 11,786 elements and 10,651 nodes. A harmonic pressure load with unit amplitude was exerted on the underside of the sandwich panel to perform a simulation of the incident acoustic wave load. The density parameters of the acoustic medium were set at *ρ* = 1.2 kg/m^3^, while the sound velocity was *c* = 343 m/s, and the volumetric modulus was *κ* = 141,179 N/m^2^. The solver utilized for the analysis is the Lanczos eigenvalue solver, which facilitates linear perturbation frequency analysis to determine the first ten natural frequencies. A steady-state dynamics approach was employed to calculate the STL curve of the structural acoustic finite element model across a frequency domain of 1 to 1000 Hz, ensuring that analysis pervaded into the stiffness region and well into the resonance region. The materials used in the referenced model are detailed in Table 2.

### 3.2. Assumptions of the Simulation Model

It is necessary to declare the limitations of this study. The main mechanism of sound propagation in a structure is determined by the bending mode. Among the bending, longitudinal, and torsional modes, only the bending mode causes the main normal velocity in surrounding air. Therefore, a two-dimensional model was used to investigate the structural acoustic behavior of the sandwich panel.

Structural damping is a key parameter that effectively reduces the surface displacement of the structure within the resonance regions, which results in an improvement in STL in these areas. However, considering that the main focus of this study was the influence of structural and geometric parameters on STL behavior, the damping effect of the aluminum was neglected in this study. A tie constraint was applied at the interface between the air and the upper surface of the panel to ensure contact and enforce continuity of the normal velocity condition.

To simplify the complexity of the acoustic simulation, the incident wave was specified as being perpendicular, representing a worst-case head-on transmission.

The sandwich panel was constrained at both sides using pinned boundary conditions, ensuring that all parts of the panel were perfectly bonded together. These boundary conditions closely resemble the actual mounting conditions of sandwich panels in reverberation rooms for practical STL measurements. Additionally, absorptive impedance boundary conditions were exerted on the circular outer surface.

Meanwhile, the quantification of numerical uncertainty and special sensitivity analysis caused by processing tolerances and material discreteness had not been considered.

### 3.3. Mesh Convergence Test

**Figure 4:**
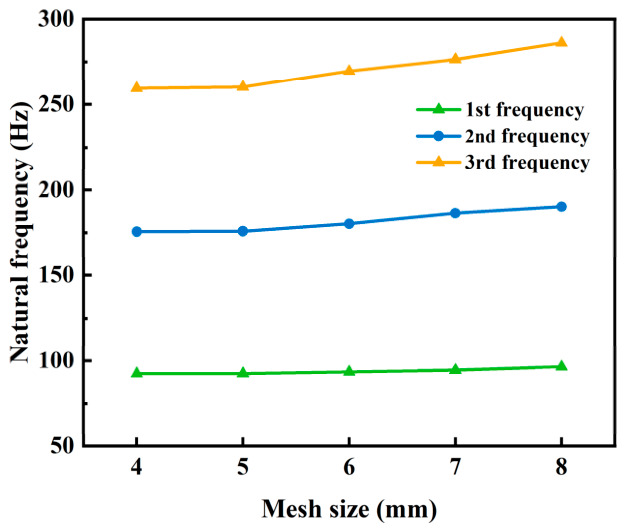
Correlation between the first three natural frequencies of M1 and mesh size.

To evaluate the impact of the mesh element size regarding the numerical results, a mesh convergence investigation was carried out with a range of mesh sizes (4, 5, 6, 7, and 8 mm) to ascertain the optimal grid dimensions for the numerical investigation. The initial three natural frequencies of the regular hexagonal metallic sandwich panel were analyzed for different mesh sizes, as illustrated in Figure 4. The curves in the figure represent the variations in the first, second, and third natural frequencies with the grid size, respectively. It was observed that as the mesh size decreased, the first three frequencies consistently converged. Upon comparison, the simulation results obtained with mesh sizes of 4 mm and 5 mm revealed no significant discrepancies. Consequently, to minimize computational effort while ensuring accuracy of the simulations, a total mesh size of 5 mm was adopted for the subsequent analyses.

### 3.4. Verification of the Finite Element Model

#### 3.4.1. Validation of the Natural Frequencies

**Figure 5:**
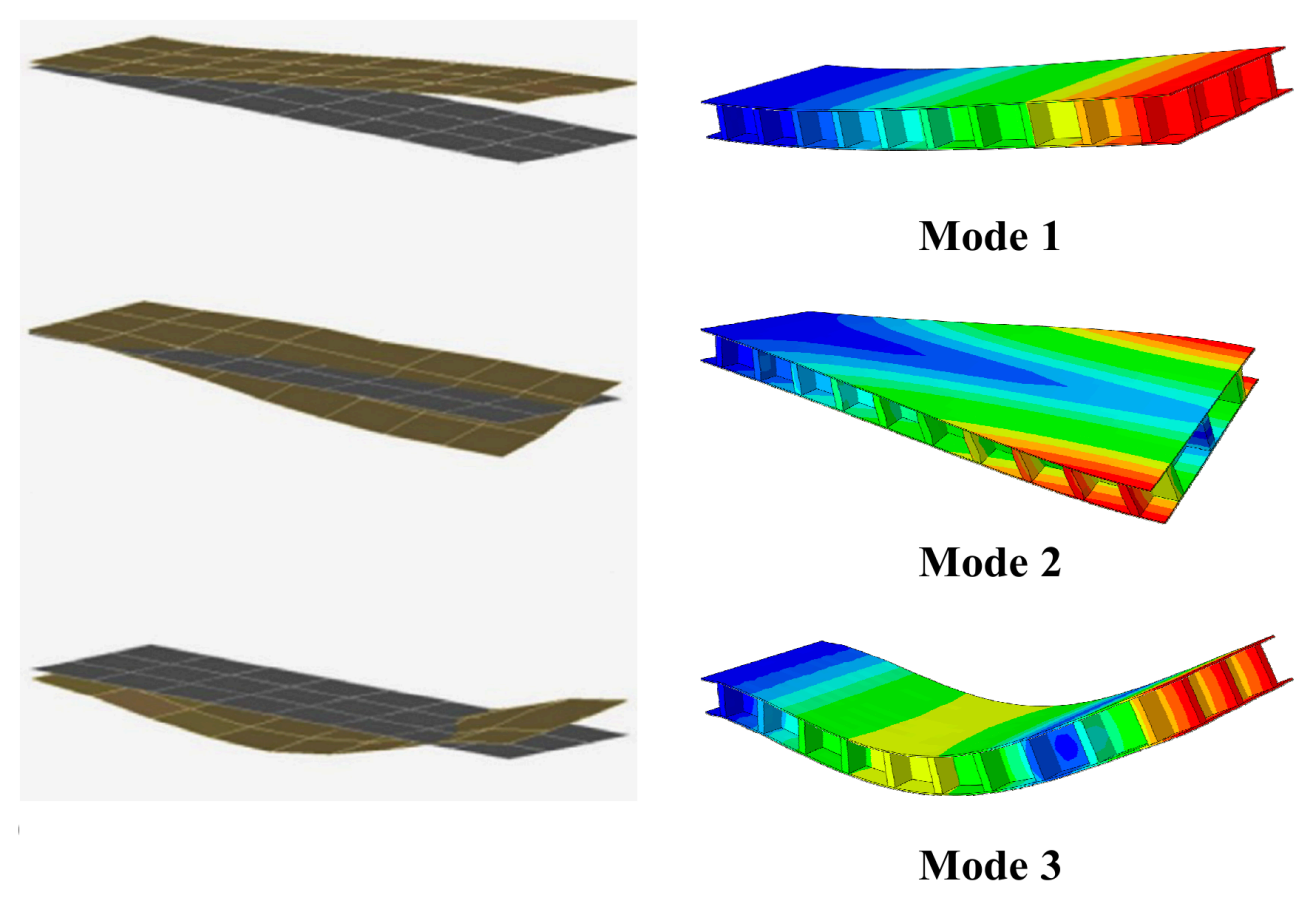
Mode shapes of experimental tests and FE simulations [49]. (The normalized relative displacement amplitude is represented by the color of the cloud map. The vibration deformation amplitude is highest in the red area and lowest in the blue area; Modal vibration mode only reflects the natural vibration mode of the structure and does not represent the stress level).

Considering that the hierarchical honeycomb is a newly introduced structure, there are no physical specimens for experiments. To prove the reliability of this simulation model, the vibration model of the square honeycomb sandwich panel was first simulated and compared with the test results by Wang [48]. Reference [48] conducted modal tests on sandwich panels and obtained the first three natural frequencies and corresponding vibration modes of the sandwich panels. To validate the reliability of the model, structural features, material parameters, and environmental conditions were aligned with those specified in the literature [48]. As shown in Table 3 and Figure 5, it was observed that the maximum discrepancy between the numerical simulations and experimental findings did not exceed 10%, indicating a close correlation between the natural frequencies derived from numerical simulations and those obtained experimentally. Considering that the test environment was not ideal, some discrepancies between the measured results and the simulated outcomes were anticipated. The results affirm that the numerical simulations possess sufficient accuracy for modeling and assessing the natural frequencies of the hierarchical sandwich panels proposed in this study.

#### 3.4.2. Verification of Transmission Loss

**Figure 6:**
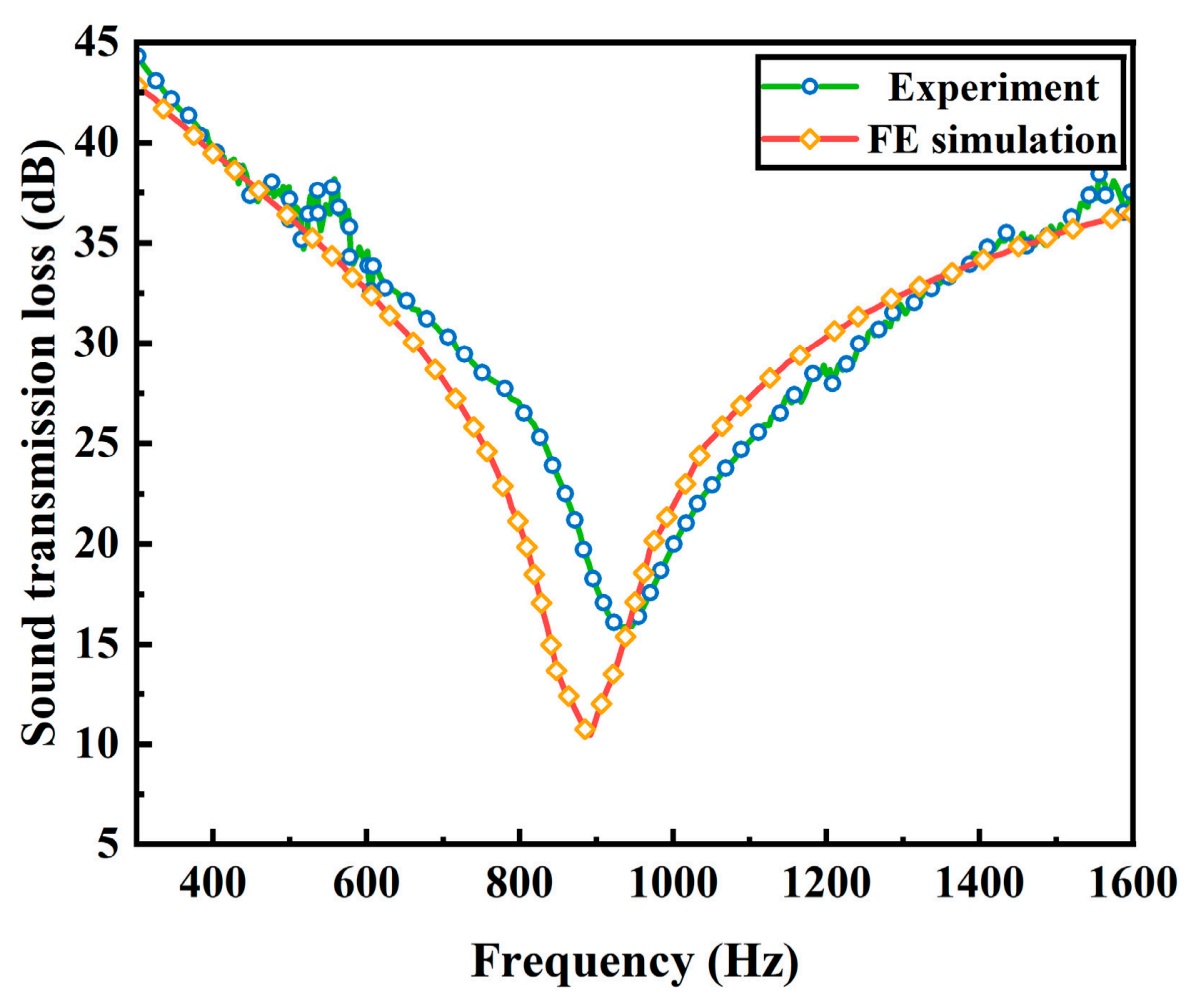
Validation analysis of experimental test [50] and finite element simulation.

In the literature [50], a square impedance tube was employed as the primary tool for sample testing. The core principle of the experimental setup was briefly summarized as detailed below: an automated signal analyzer controlled by a computer (BSWA-MC 3242 A, Beijing BSWA Technology Co., Ltd., Beijing, China) emits plane sound waves, which are subsequently amplified by a power amplification device (BSWA-PA 50) covering a frequency spectrum from 45 to 1600 Hz. The acoustic waves then infiltrated the impedance tube through a loudspeaker situated on the left side of the tube. A pair of microphones (BSWA-MPA 416) was positioned adjacent to the sample for data acquisition, which was relayed back to the signal analyzer. Ultimately, the experimental data for the STL recorded for the specimen were computed using assessment software (VA-Lab, https://www.bswa.com.cn/?p=573&a=view&r=613&city_name=, accessed on 26 July 2026). The testing configuration of this study maintained the same structural characteristics and material parameters as those noted in the reference literature to ensure model reliability. As depicted in Figure 6, one can see that the trends and trough positions of the STL curves obtained through testing methodologies closely align with those derived through theoretical approaches. Notably, the first resonant frequency differs by 22.5 Hz between the predicted (900 Hz) and experimental (922.5 Hz) outcomes, while the amplitude discrepancies in the STL curves ranged from 1 to 5 dB. These discrepancies can be primarily attributed to constraints or gaps present in the region between the test sample and the impedance tube walls, which yield imperfect contact conditions and consequently diminish the accuracy of the measurements related to acoustic performance. Nevertheless, the overall trends between the two methodologies exhibit significant similarity. In conclusion, the sound insulation simulations demonstrated sufficient accuracy, thereby validating the use of finite element methods for investigating the acoustic performance of hierarchical honeycombs.

## 4. Results and Discussion

### 4.1. Comparison Between the Hierarchical Honeycomb Structure and Hexagonal Honeycomb Structure

#### 4.1.1. Natural Frequencies

**Figure 7:**
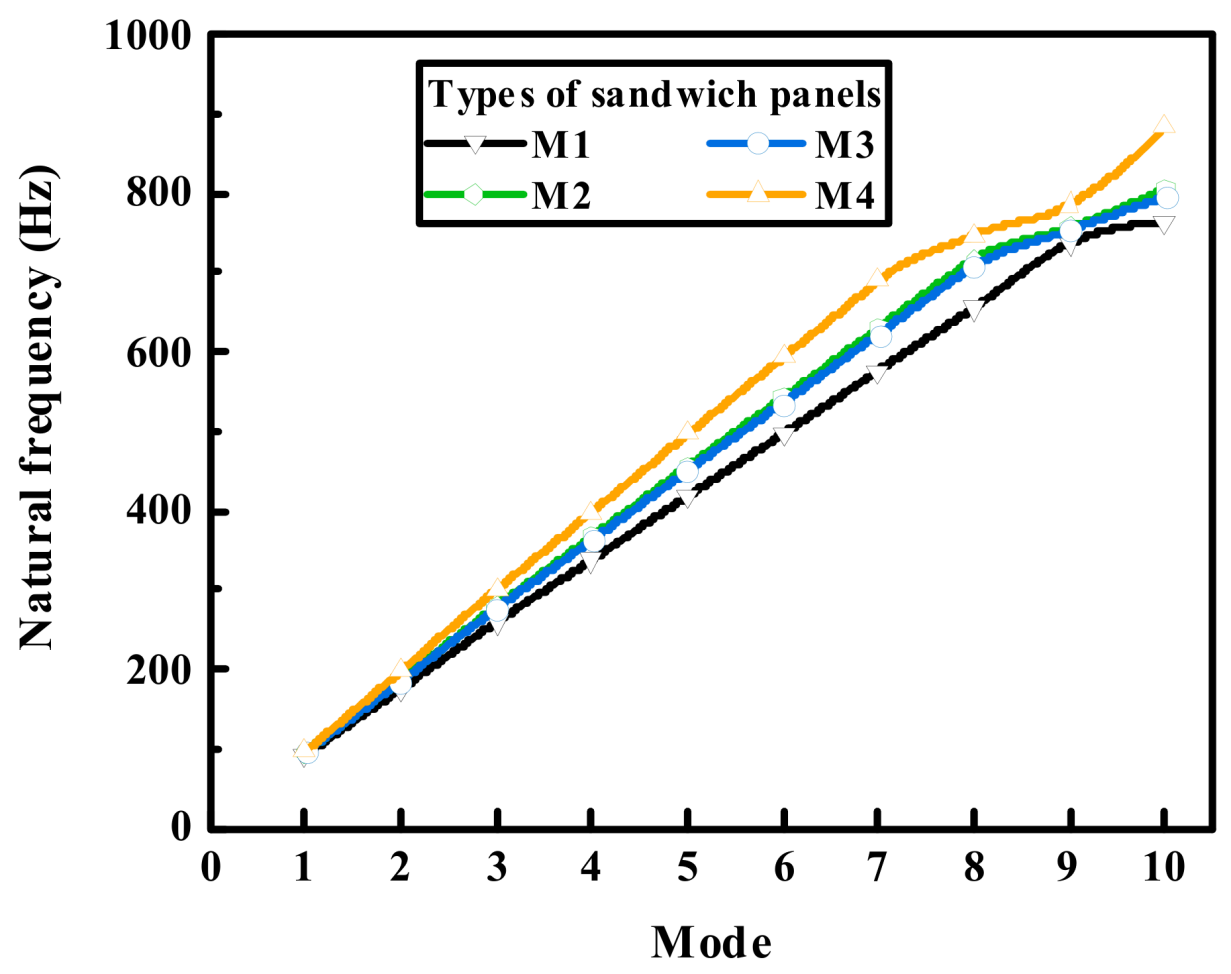
The first ten natural frequencies of panels M1, M2, M3, and M4 with geometric parameters in Table 1.

For the four different configurations of sandwich structures mentioned in Table 1, the consequences resulting from vertex substitution impacting the vibrational attributes of the sandwich panels were investigated, with the first ten natural frequencies presented in Table 4. As illustrated in Figure 7, the natural frequency of the sandwich panel M1 was lower than those of the other three configurations with substituted vertices. This result was attributed to the enhanced bending resistance resulting from the introduction of hierarchical characteristics. Higher stiffness can elevate the overall resonant frequency, avoiding low-frequency resonance-induced sound insulation dips. A comparative analysis of the simulation results for sandwich panels M1, M2, and M3 revealed that the curves for M2 and M3 nearly coincide, while M3 exhibited a notable improvement. In other words, the sandwich panels with circular and regular hexagonal vertices displayed negligible differences in natural frequencies, while the hierarchical sandwich panel with triangular vertices demonstrated the highest natural frequency.

#### 4.1.2. Sound Transmission Loss

**Figure 8:**
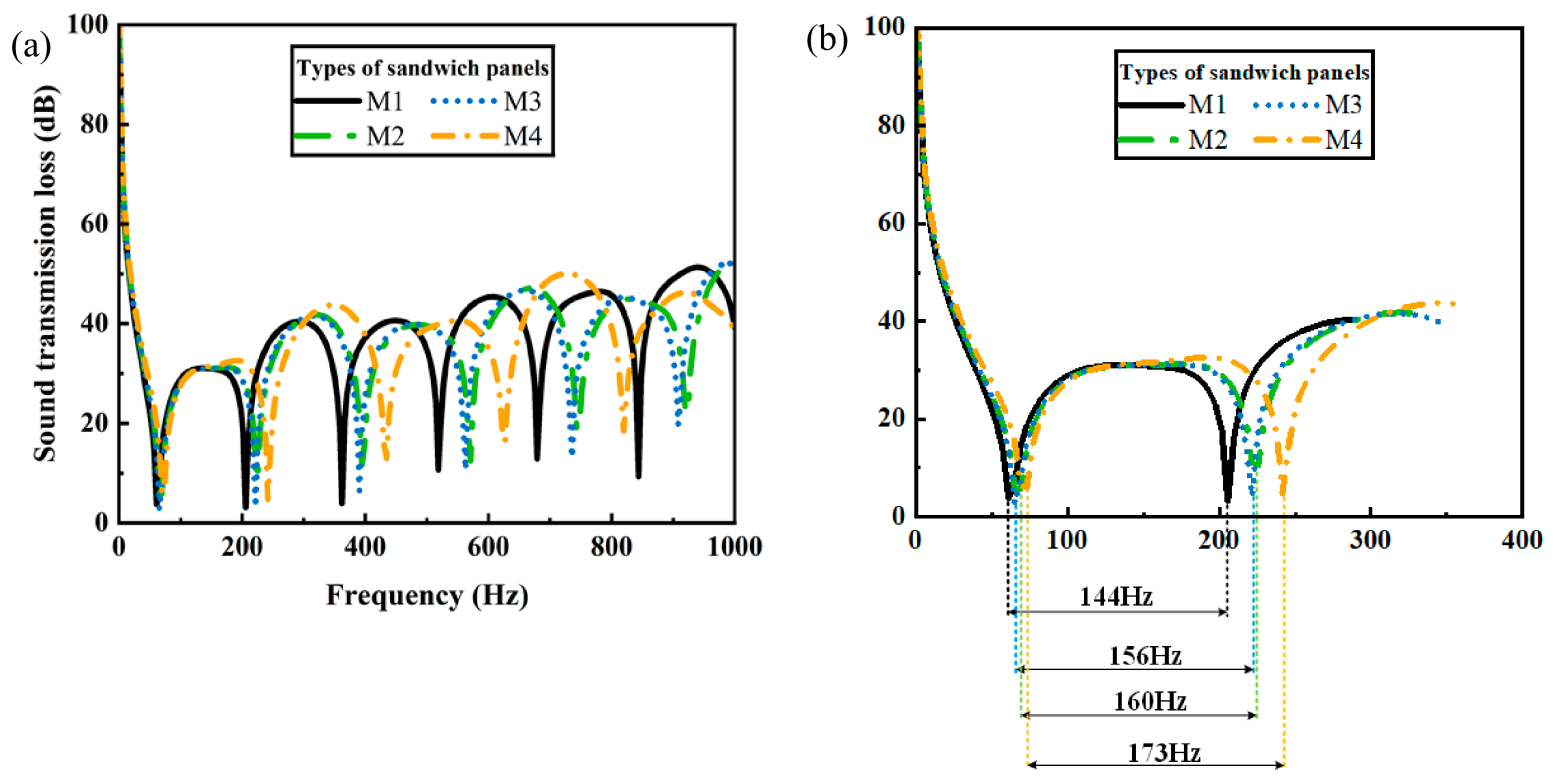
Comparison of sound transmission loss (STL) performance of panels M1, M2, M3, and M4 with geometric parameters in Table 1: (**a**) full frequency range (0–1000 Hz); (**b**) close-up view (0–200 Hz).

This study employed a steady-state dynamic response analysis to output the sound pressure values at each node and subsequently calculate the STL of the panels. The process for STL calculation and transmission curve plotting is elaborately depicted in Figure 8. The acoustic design was created utilizing finite element software ABAQUS 2022, where material properties were defined and where inherent frequencies (Hz), boundary conditions, sound impedance, and incident sound waves were set, followed by mesh discretization. The sound pressure values for the hierarchical sandwich panels were obtained through the steady-state dynamic analysis procedure, and the STL of the panels was computed using Equation (10). For the sake of simplification, STL is expressed in decibels, which represents the ratio of the incident sound pressure to the transmitted sound pressure through the panel [11,51].

(10) $$STL=10{log}_{10}\left|\frac{p_{i}^{2}}{p_{t}^{2}}\right|$$

where $p_{i}$ and $p_{t}$ represent the quadratic mean sound pressure measured on the sides related to the incident and transmitted sound, respectively. The formulas for calculating $p_{i}$ and $p_{t}$ are as follows:

(11) $$p_{i}^{2}=\frac{1}{M}\sum_{k=1}^{M}\;p_{k}^{2},p_{t}^{2}=\frac{1}{N}\sum_{k=1}^{N}\;p_{k}^{2}$$

In the equation, $p_{k}$ represents the pressure value recorded at the *k*-th nodal point on the surface of the sandwich panel. *M* and *N* denote the node density on the side of incidence and the transmitted side, respectively. The pressure at the nodes located on the incident surface was configured to 1 Pa, while the pressure on the transmission surface was obtained by extracting data from the nodes on the air layer closely attached to the panel using finite element software.

By analyzing Figure 8, it can be observed that based on the modal results, each natural frequency corresponds to a sound insulation valley on the STL curve. As the structural vertices change, the STL values of the hierarchical sandwich panels M2, M3, and M4 in the stiffness control region were slightly higher than that of the original panel M1. In the resonance region, the hierarchical sandwich panels with circular, hexagonal, and triangular vertices exhibited fewer sound transmission notches compared to the original panel M1. This finding suggested that the modifications to the structure reduced the possibility of resonance, thereby enhancing acoustic performance. Furthermore, as displayed in Figure 8, the STL values of the panels with circular and hexagonal vertices were nearly identical, with only a 4 Hz difference in the spacing between the first downward points. Conversely, the panel with triangular vertices displayed a significantly higher STL than those of M1, M2, and M3, accompanied by a much greater distance between its initial downward points. This superior performance can be attributed to the enhanced rigidity and resonance characteristics of the triangular hierarchical sandwich panel. The spacing between the downward points further indicated that the triangular configuration offers greater flexibility in sound insulation performance compared to the other structures. Therefore, the hierarchical sandwich panel featuring triangular vertices exhibited superior sound insulation capabilities.

#### 4.1.3. Distribution of Sound Pressure Levels

In addition to the research on sound insulation mentioned above, a graphical analysis of the distribution of transmitted sound pressure in the sound field is also provided. The sound pressure level (SPL) was utilized to outline the radiated sound pressure [52]. SPL is defined as follows:

(12) $$SPL=20\times {log}_{10}\frac{p_{e}}{p_{ref}}$$

In the equation, $p_{ref}$ represents the reference standard pressure, while $p_{e}$ denotes the sound pressure.

**Figure 9:**
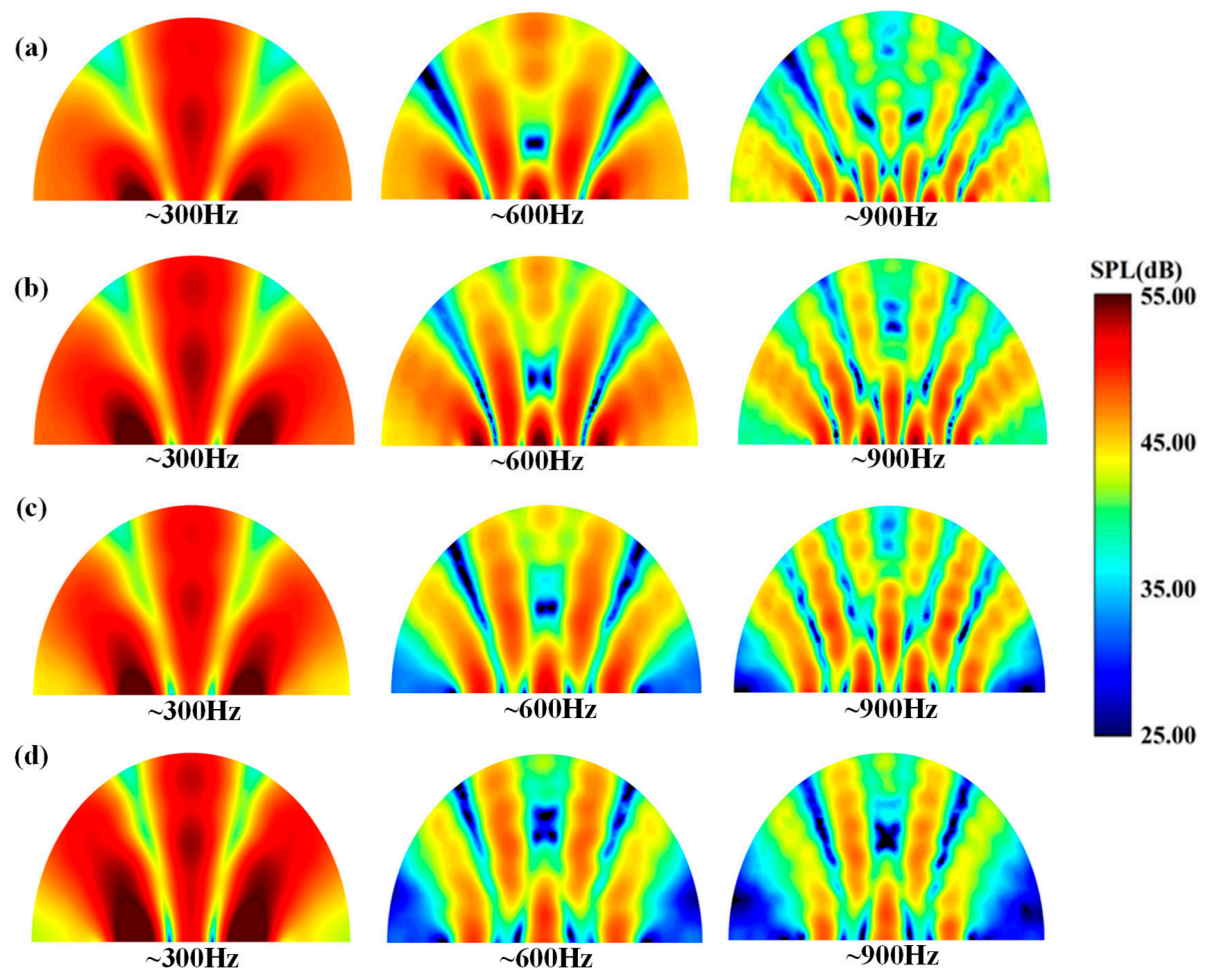
SPL distribution of panels M1, M2, M3, and M4 with geometric parameters in Table 1 at 300, 600, and 900 Hz: (**a**) M1; (**b**) M2; (**c**) M3; (**d**) M4.

From analyzing Figure 9, it was observed that for the various configurations of the sandwich panels, the SPL values in the air area adjacent to the panels at lower frequency ranges significantly exceeded 55 dB. Conversely, at higher frequency ranges, the SPL values in the air domain at greater distances consistently remained below 25 dB. The original panel structure M1 exhibited high SPL values occupying a substantial area of the air domain. It is noteworthy that the radiated SPL values of the original panel were always higher than those of the hierarchical honeycomb panels, with M2 and M3 displaying minimal differences in their SPL radiation values, aligning with the results obtained from the aforementioned STL analysis.

From the perspective of acoustic impedance, the introduction of hierarchical characteristics into honeycomb topology can create more impedance discontinuity interfaces, thereby enhancing the proportion of acoustic wave interface reflection. From the perspective of elastic wave propagation paths, the introduction of hierarchical characteristics can extend the distance of wave propagation within the core layer. Through scattering and internal structural damping, the transmitted acoustic energy is significantly attenuated.

Additionally, the hierarchical sandwich panel with triangular vertices demonstrates lower SPL values compared to those with circular and regular hexagonal vertices, with the attenuation increasing with frequency. In summary, the hierarchical structure with triangular vertices demonstrates superior sound insulation capabilities, while M1 has the worst sound insulation effect due to its low stiffness and direct wave propagation paths.

### 4.2. Discussion

#### 4.2.1. The Influence of Vertex Size

**Figure 10:**
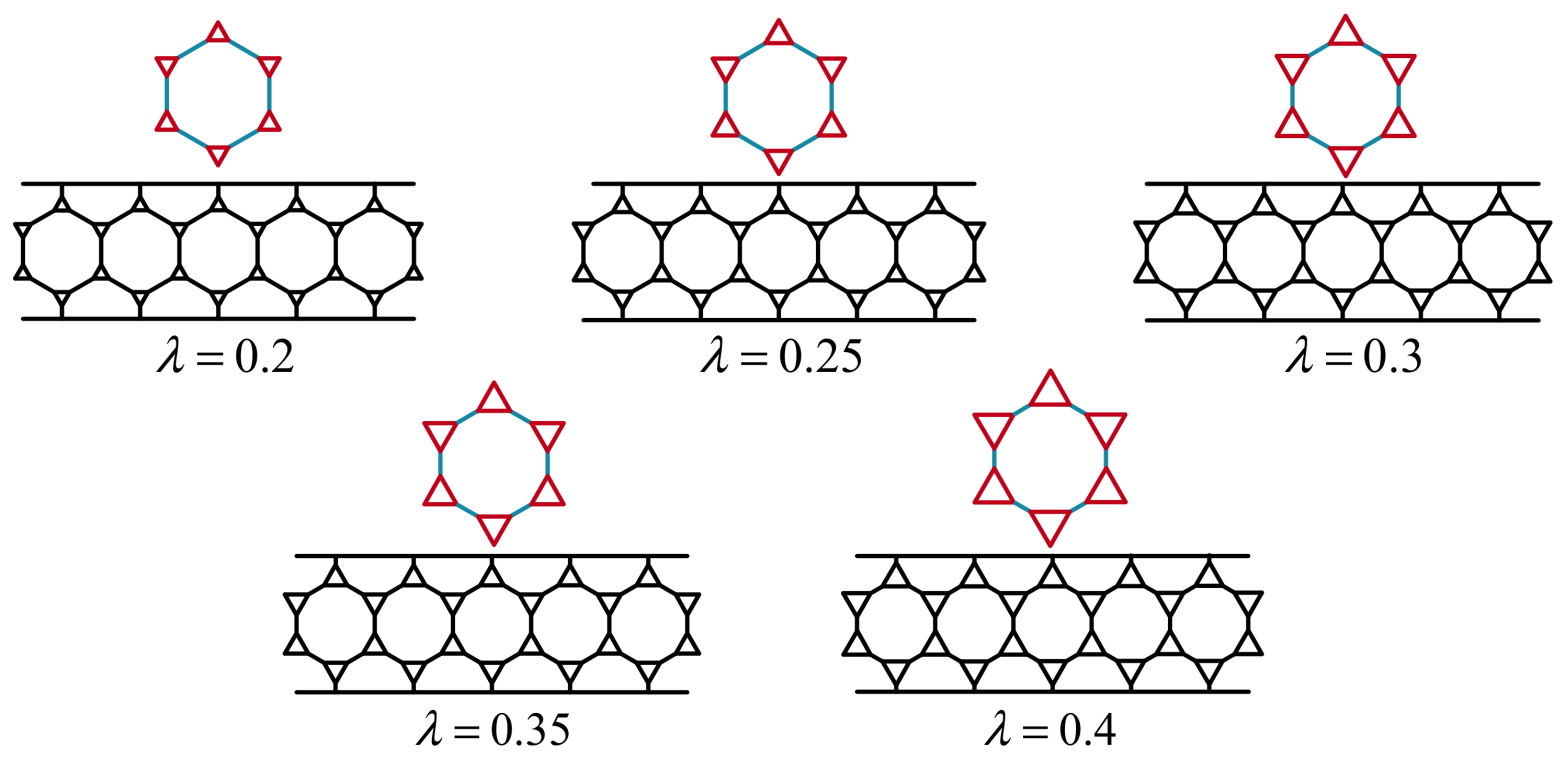
Hierarchical honeycombs with different structural hierarchical features λ.

The above finite element analysis results demonstrated that substituting the vertices of the hexagonal structure with alternative geometries can significantly enhance the vibration attenuation and sound insulation properties, with the triangular vertex hierarchical honeycomb structure emerging as the most advantageous configuration. Based on the aforementioned results, this section focuses on the hierarchical honeycomb with triangular vertices and discusses the impact of varying the size of the honeycomb vertices on vibration reduction and sound insulation capabilities. A comprehensive examination of the triangular vertex hierarchical honeycomb structure was undertaken, focusing on the fluctuations of the hierarchical parameter λ within the range of 0.2 to 0.4, employing an incremental step size of 0.05. The unit cells and other structural dimensions of hierarchical honeycombs with different λ are shown in Table 5. Figure 10 visually represents the hierarchical honeycomb sandwich panels with distinct hierarchical parameters.

**Figure 11:**
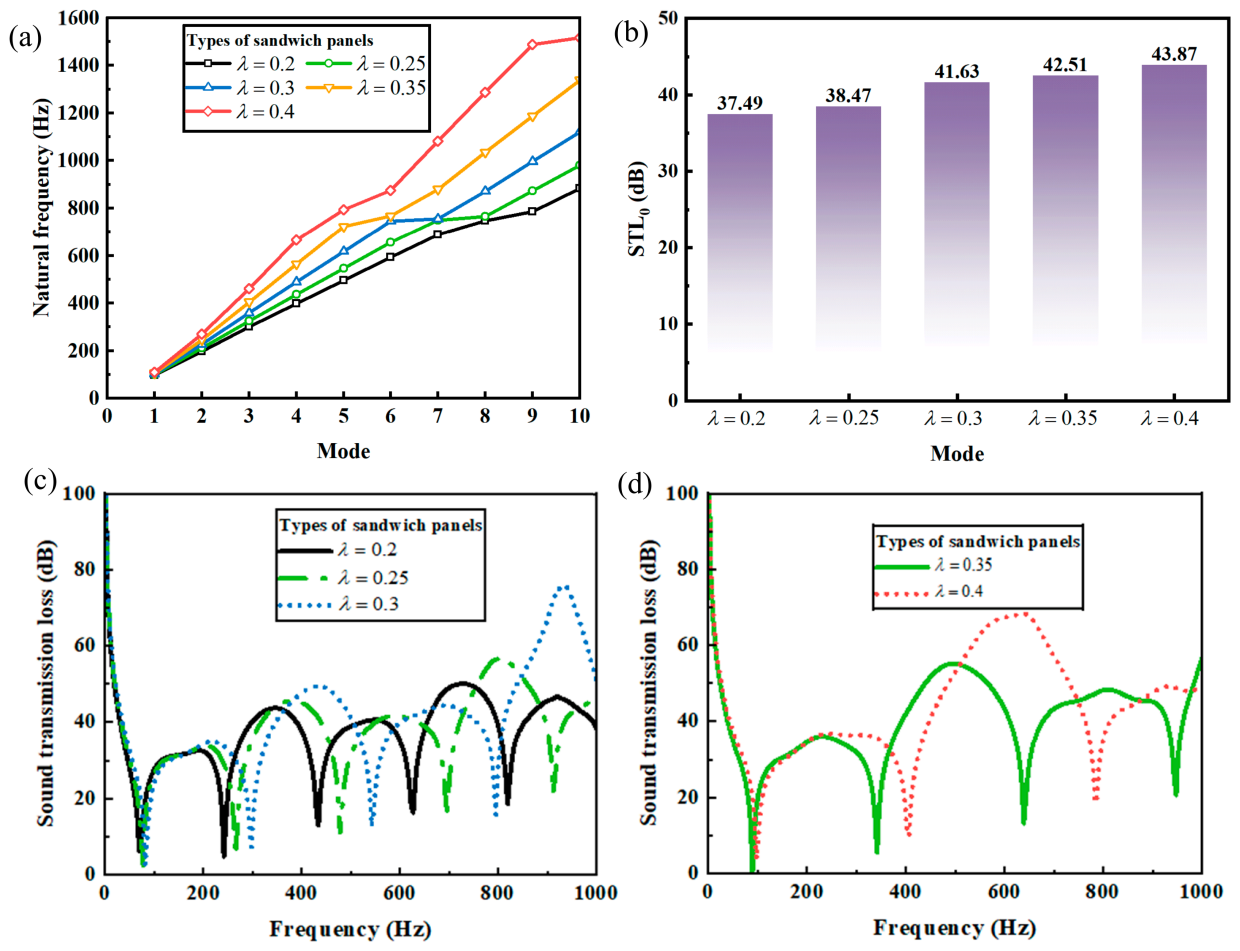
The influence of hierarchical parameter λ for hierarchical honeycombs with unit cells 1 × 40 and $h_{0}$ = 28.87 mm: (**a**) natural frequency; (**b**) the average transmission loss; (**c**) STL curves for λ = 0.2, 0.25 and 0.3; (**d**) STL curves for λ = 0.35 and 0.4.

**Figure 12:**
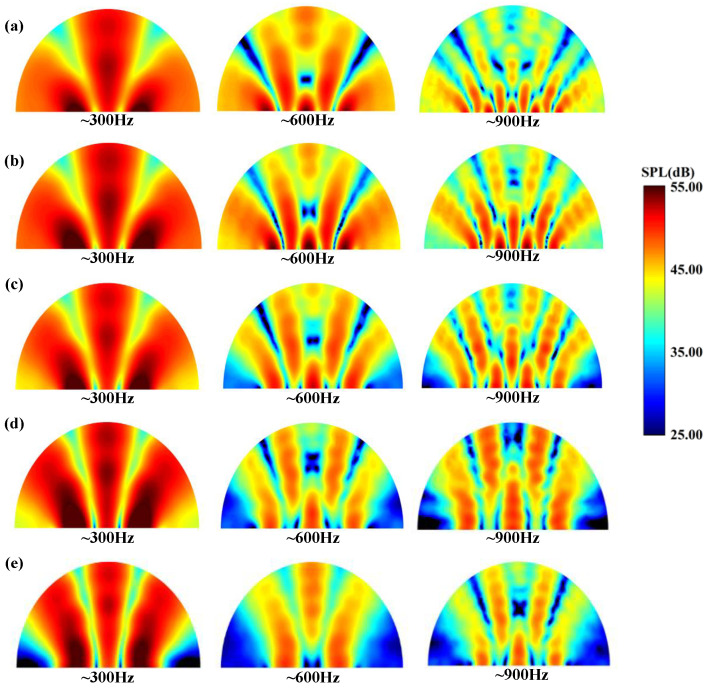
The influence of hierarchical parameter λ on the SPL distribution for hierarchical honeycombs with unit cells 1 × 40 and $h_{0}$ = 28.87 mm at 300 Hz, 600 Hz, and 900 Hz: (**a**) λ = 0.2; (**b**) λ = 0.25; (**c**) λ = 0.3; (**d**) λ = 0.35; (**e**) λ = 0.4.

The simulation results are comprehensively depicted in Figure 11. In Figure 11a, it is evident that a positive correlation exists between the frequency and hierarchical parameter λ, suggesting that an enhancement in λ significantly augments the transmission loss associated with the natural frequency. Figure 11b illustrates a bar chart of the average STL, denoted as ${STL}_{o}$. In comparison to λ=0.2, the ${STL}_{o}$ values for λ = 0.25, 0.3, 0.35, and 0.4 exhibited increases of 2.6%, 11%, 13.4%, and 17.2%, respectively. Moreover, Figure 11c presents the STL curves corresponding to different λ values. Notably, as λ escalates, the curves in the resonance region indicated a shift in the resonant valleys toward higher frequencies, accompanied by a corresponding elevation in the transmission loss values at the peaks. Furthermore, Figure 12 reveals a marked acceleration in the attenuation rate of the SPL with an increasing λ. Thus, the preceding analysis underscored the conclusion that an increment in λ effectively enhanced the vibration damping capabilities and soundproofing efficiency of the hierarchical honeycomb structure.

#### 4.2.2. The Influence of Unit Size

Previous analyses had indicated that, under the condition of maintaining consistent mass, the enhancement in sound insulation performance brought about by a hierarchical design was substantial. However, the correlation of unit size with the sound insulation performance of new hierarchical honeycomb sandwich panels remained uncertain. Therefore, while ensuring equal mass, the impact of unit size regarding sound insulation performance will be studied. To optimize computational efficiency, the transmission loss curves for hierarchical honeycomb sandwich beams with $h_{0}$ values of 28.87 mm and 14.43 mm were computed for λ=0.2, 0.3, and 0.4. The unit cells and other structural dimensions of hierarchical honeycombs with different unit sizes are shown in Table 6.

**Figure 13:**
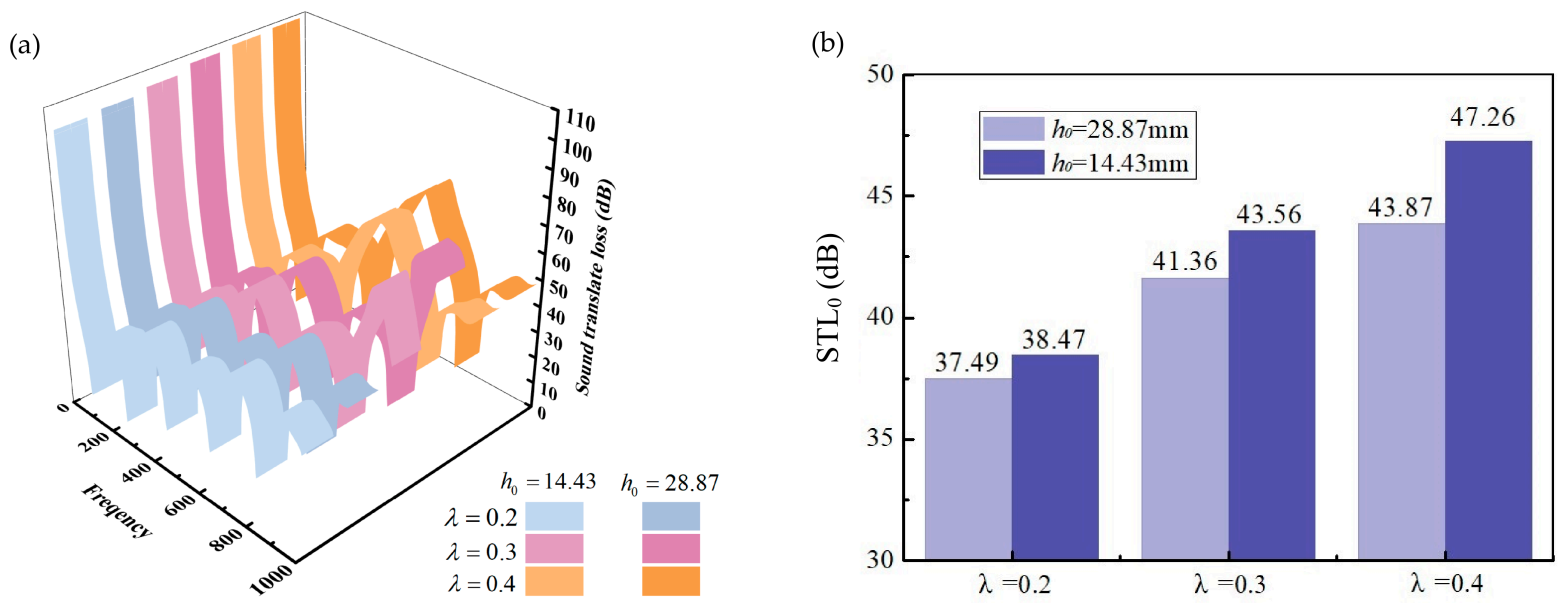
The sound insulation performance for hierarchical honeycombs with different unit sizes, including six structures: λ= 0.2, $h_{0}=28.87\;\mathrm{mm}$; λ= 0.2, $h_{0}=14.43\;\mathrm{mm}$; λ= 0.3, $h_{0}=28.87\;\mathrm{mm}$; λ= 0.3, $h_{0}=14.43\;\mathrm{mm}$; λ= 0.4, $h_{0}=28.87\;\mathrm{mm}$; λ= 0.4, $h_{0}=14.43\;\mathrm{mm}$: (**a**) STL curves; (**b**) average sound transmission loss.

**Figure 14:**
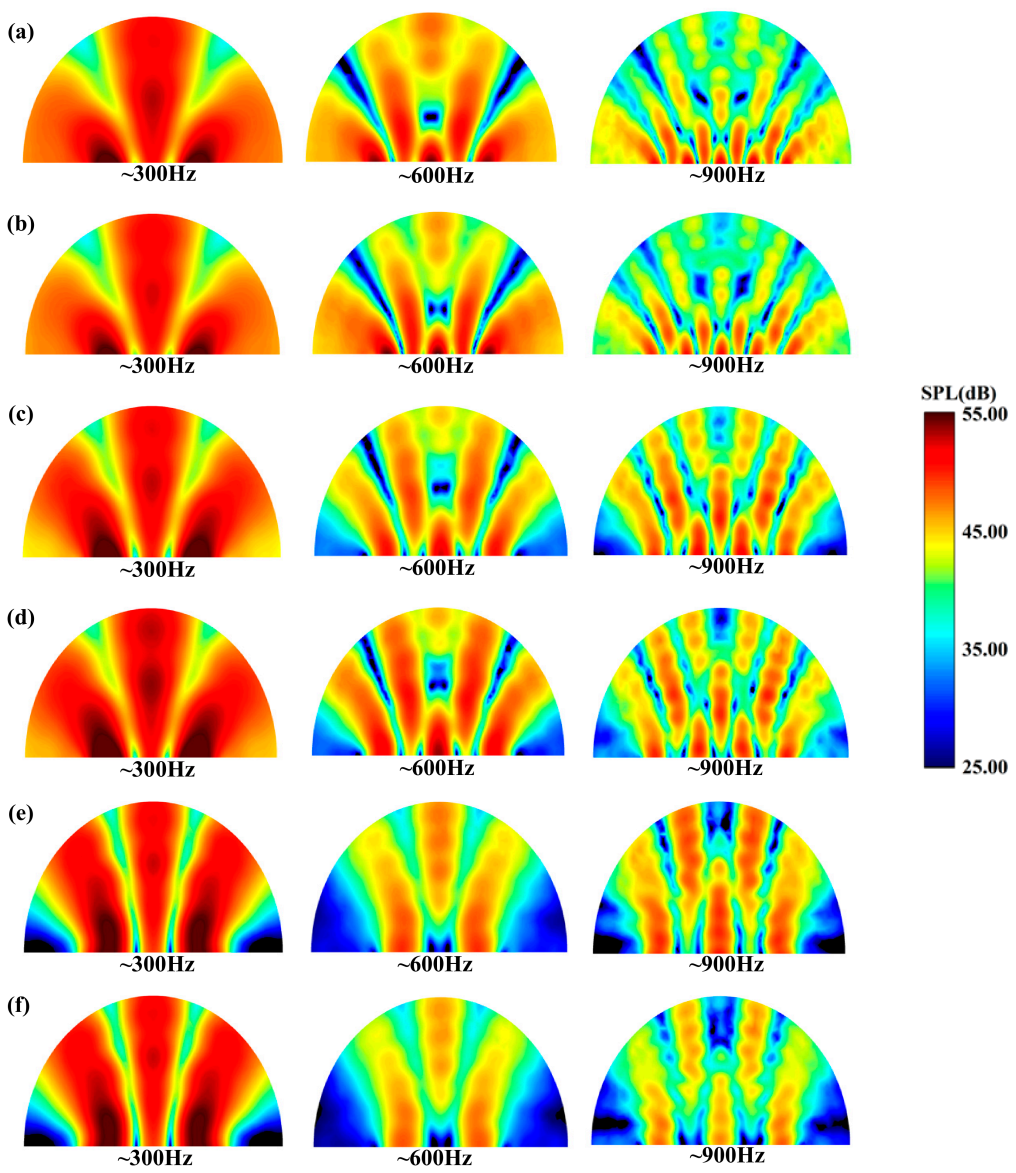
The SPL distribution for hierarchical honeycombs with different unit sizes at 300 Hz, 600 Hz, and 900 Hz: (**a**) λ= 0.2, $h_{0}=28.87\;\mathrm{mm}$; (**b**) λ= 0.2, $h_{0}=14.43\;\mathrm{mm}$; (**c**) λ= 0.3, $h_{0}=28.87\;\mathrm{mm}$; (**d**) λ= 0.3, $h_{0}=14.43\;\mathrm{mm}$; (**e**) λ= 0.4, $h_{0}=28.87\;\mathrm{mm}$; (**f**) λ= 0.4, $h_{0}=14.43\;\mathrm{mm}$.

It is apparent from Figure 13 that when $h_{0}$ is set at 14.43 mm, the STL curves are elevated in comparison to those at $h_{0}=28.87$ mm, irrespective of the λ values. The curves in the resonance region demonstrated that the valleys shifted toward the higher frequency domain. Specifically, the average sound transmission loss ${STL}_{o}$ for $h_{0}=14.43$ mm exhibited increases of 2.6%, 5.1%, and 7.7% for λ = 0.2, 0.3, and 0.4, respectively, compared to $h_{0}=28.87$ mm. Furthermore, as illustrated in Figure 14, when $h_{0}$ is at 14.43 mm, the area with SPL values exceeding 55 dB diminishes while the area with SPL values falling below 25 dB expands. Overall, research has shown that reducing the unit size slightly improved the sound insulation performance of the hierarchical honeycomb.

#### 4.2.3. The Influence of Vertex Angle

**Figure 15:**
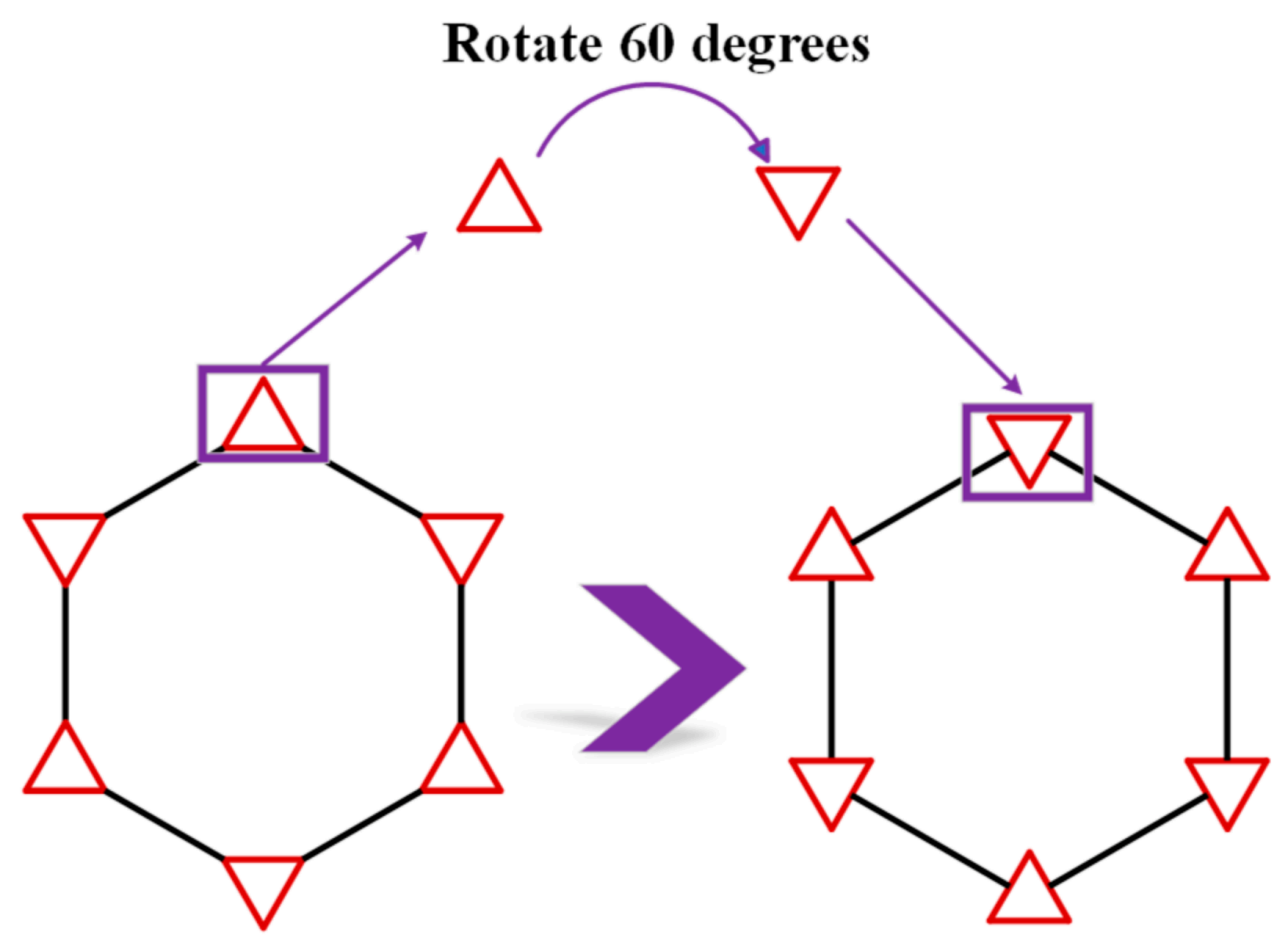
Honeycomb unit after vertex rotation of 60 degrees.

In order to conduct a more in-depth investigation into the repercussions of triangular vertices on the sound insulation performance of hierarchical structures, λ = 0.2, 0.3, and 0.4 were chosen as examples. By rotating the triangular vertex by 60 degrees, a novel hierarchical structure was obtained, as illustrated in Figure 15, with specific parameters detailed in Table 7.

**Figure 16:**
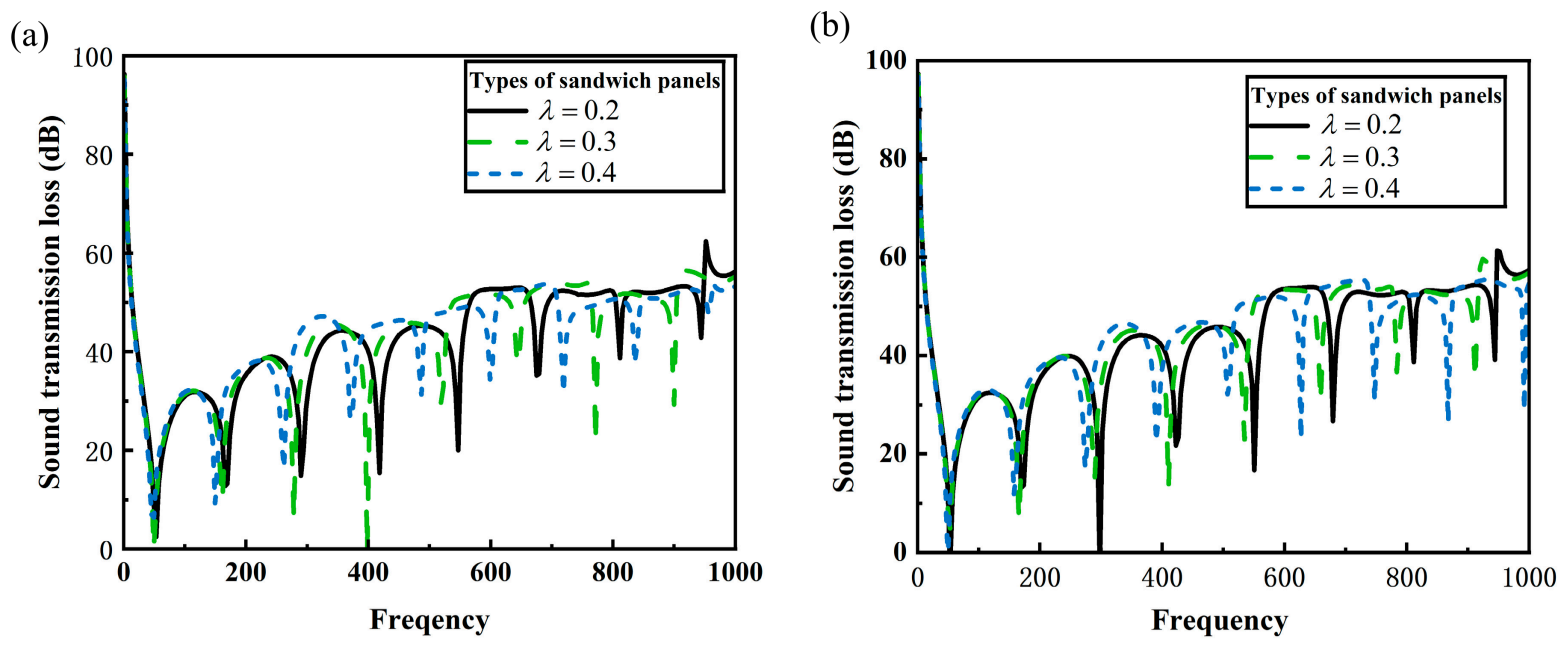
The STL curves of hierarchical honeycombs with different λ after a vertex rotation of 60°: (**a**) hierarchical honeycomb with unit cells 1 × 40 and $h_{0}$ = 28.87 mm; (**b**) hierarchical honeycomb with unit cells 2 × 80 and${\;h}_{0}$ = 14.43 mm.

**Figure 17:**
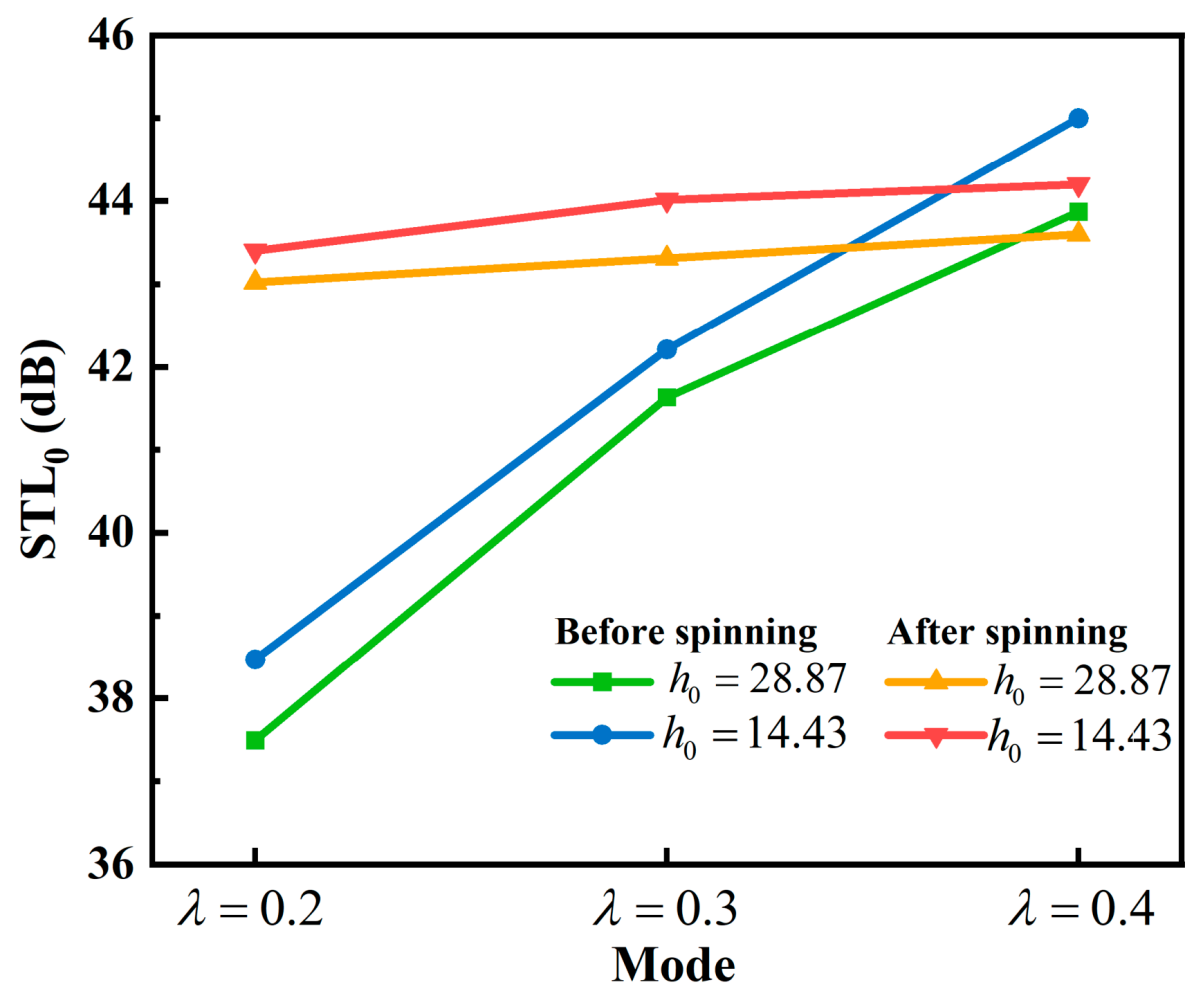
The ${STL}_{o}$ of hierarchical honeycombs before and after a vertex rotation of 60° (the green and yellow line data represent hierarchical honeycomb with unit cells 1 × 40 and $h_{0}$ = 28.87 mm; the blue and red line data represent hierarchical honeycomb with unit cells 2 × 80 and $h_{0}$ = 14.43 mm).

The STL curves depicted in Figure 16 reveal that after the triangular vertex was rotated by 60 degrees, the valleys of the STL curves shifted toward lower frequencies with the increase of λ, while the number of sound insulation notches also increased accordingly. Figure 17 reveals that the ${STL}_{o}\;$of hierarchical honeycomb will increase with the increase of λ, regardless of whether the vertices are rotated or not. After the vertex is rotated, the trend of ${STL}_{o}$ increasing with λ will slow down. The comparison of ${STL}_{o}$ before and after vertex rotation reveals that when λ is 0.2 and 0.3, the rotation of the vertex triangle can lead to an increase in ${STL}_{o}$, while the opposite phenomenon occurs when λ is 0.4. Specifically, when $h_{0}$ was set at 28.87 mm, the ${STL}_{o}$ values for the hierarchical honeycomb after vertex triangle rotation showed increases of 14.8% and 4% in comparison to the pre-rotation structures for λ = 0.2 and λ = 0.3, respectively. However, for λ = 0.4, the ${STL}_{o}$ values for post-rotation structures fell below those recorded prior to the rotation. When $h_{0}$ was set at 14.43 mm and 28.87 mm, respectively, the ${STL}_{o}$ values for the rotated structures showed a decrease of 1.83% and 0.64%, respectively.

The above analysis indicates that the influence of vertex triangle rotation on the STL of hierarchical honeycomb is closely related to the hierarchical parameters. When the hierarchical parameters are small, performing vertex triangle rotation on the hierarchical honeycomb is beneficial for improving its sound insulation performance.

## 5. Conclusions

The principal aim of this study is to put forward a hierarchical honeycomb model and conduct a comprehensive investigation of the vibration and acoustic performance of this novel structure. A numerical simulation technique was employed to evaluate the sound insulation performance of sandwich panels with hierarchical characteristics. Within the given simulation parameter range in this article, the key conclusions from this research were as follows:

(1) The proposed hierarchical honeycomb core exhibits superior vibration reduction and soundproofing effectiveness when contrasted with its conventional honeycomb, and its actual noise reduction effect still needs to be further verified by subsequent physical experiments. When the vertices were configured as regular hexagons, circles, and equilateral triangles, there was a significant augmentation of the natural frequency. The sandwich panel featuring triangular vertices demonstrated the most effective vibration reduction capability. Overall, hierarchical honeycomb enhanced a more universal sound insulation performance, and vertex triangles replacing hierarchical honeycomb exhibited stronger sound insulation capabilities.
(2) The hierarchical honeycomb with triangular vertices can augment the acoustic insulation capacity. Under the same wall thickness conditions, the ${STL}_{o}$ values for λ = 0.25, 0.3, 0.35, and 0.4 exhibited increases of 2.6%, 11%, 13.4%, and 17.2%, respectively, compared to the hierarchical honeycomb with λ=0.2. Additionally, as the value of λ increased, the vibration reduction capability improved correspondingly.
(3) The impact of unit size on vibration and sound insulation performance was significant. Regardless of the value of λ, a smaller $h_{0}$ consistently demonstrated superior sound insulation performance compared to larger $h_{0}$ dimensions. This indicated that optimizing the unit size was crucial for enhancing its acoustic behavior.
(4) The influence of vertex triangle rotation on the STL of hierarchical honeycomb is closely related to the hierarchical parameters. When λ is 0.2 and 0.3, the rotation of the vertex triangle can lead to increases in ${STL}_{o}$, while the opposite phenomenon occurs when λ is 0.4. When $h_{0}$ was set at 28.87 mm, the ${STL}_{o}$ values for the hierarchical honeycomb after vertex triangle rotation showed increases of 14.8% and 4% in comparison to the pre-rotation structures for λ = 0.2 and λ = 0.3, respectively. When the hierarchical parameters are small, performing vertex triangle rotation on the hierarchical honeycomb is beneficial for improving its sound insulation performance.

Based on the numerical calculation results, this structure has the potential for sound insulation applications in ship cabins within the target frequency band. However, the performance under actual conditions still depends on manufacturing processes, physical testing, and experimental verification. The adaptability needs to be confirmed through actual installation conditions and experimental testing in the future.

## Figures and Tables

**Table 1:** Geometric parameters of the sandwich panel (Unit: mm).

Model	Vertex Configuration	$L_{x}$	$L_{y}$	$h_{0}$	$t_{f}$	λ	t	$t_{r}$	$t_{c}$	$t_{e}$
M1	Empty	50	86.6	28.87	2.5	-	2.5	-	-	-
M2	Hexagon vertices	50	86.6	28.87	2.5	0.2	-	1.78	-	-
M3	Circular vertices	50	86.6	28.87	2.5	0.2	-	-	1.74	-
M4	Triangle vertices	50	86.6	28.87	2.5	0.2	-	-	-	2.09

**Table 2:** Material attributes.

	Mass Density ($kg/m^{3}$)	Young’s Modulus (MPa)	Poisson’s Ratio
Aluminum	2700	71,900	0.3

**Table 3:** First three natural frequencies (Hz) of experimental [48] and numerical results.

Order	Square Honeycomb		Cross Honeycomb	
	Experimental Results [48]	Numerical Results	Experimental Results [48]	Numerical Results
1	210	215	162	175
2	781	841	471	514
3	985	1039	583	631

**Table 4:** The first ten natural frequencies of panels M1, M2, M3, and M4 with geometric parameters in Table 1.

Mode	1	2	3	4	5	6	7	8	9	10
M1	92.53	175.72	259.40	338.23	418.26	496.37	576.45	655.28	736.48	761.99
M2	95.50	186.98	279.31	367.06	455.49	542.01	629.88	716.28	756.02	804.35
M3	94.95	185.23	276.31	362.83	450.09	535.42	622.14	707.34	753.15	794.24
M4	97.08	197.77	300.11	398.19	496.13	592.35	689.36	747.30	785.25	882.34

**Table 5:** Parameters of hierarchical honeycombs (with unit cells 1 × 40 and $h_{0}$ = 28.87 mm) based on different hierarchical parameter λ.

Hierarchical Honeycomb		
λ	$h_{e}$ (mm)	$t_{e}$ (mm)
0.2	5.77	2.09
0.25	7.22	2.01
0.3	8.66	1.93
0.35	10.10	1.86
0.4	11.55	1.80

**Table 6:** Parameters of hierarchical honeycombs based on different unit sizes.

Hierarchical Honeycomb				
$h_{0}$ (mm)	Unit Cells	λ	$h_{e}$ (mm)	$t_{e}$ (mm)
14.43	2 × 80	0.2	2.89	1.18
14.43	2 × 80	0.3	4.33	1.09
14.43	2 × 80	0.4	5.77	1.02
28.87	1 × 40	0.2	5.77	2.09
28.87	1 × 40	0.3	8.66	1.93
28.87	1 × 40	0.4	11.55	1.80

**Table 7:** Parameters of hierarchical honeycombs with different units after vertex rotation.

Hierarchical Honeycomb				
$h_{0}$ (mm)	Unit Cells	λ	$h_{e}$ (mm)	$t_{e}$ (mm)
14.43	2 × 80	0.2	2.89	1.05
14.43	2 × 80	0.3	4.33	0.95
14.43	2 × 80	0.4	5.77	0.87
28.87	1 × 40	0.2	5.77	1.86
28.87	1 × 40	0.3	8.66	1.67
28.87	1 × 40	0.4	11.55	1.53

## Data Availability

The original contributions presented in this study are included in the article. Further inquiries can be directed to the corresponding author.
